# *In silico* Screening and Evaluation of the Anticonvulsant Activity of Docosahexaenoic Acid-Like Molecules in Experimental Models of Seizures

**DOI:** 10.6091/.21.1.32

**Published:** 2017-01

**Authors:** Ali Gharibi Loron, Soroush Sardari, Jamshid Narenjkar, Mohammad Sayyah

**Affiliations:** 1 Department of Physiology and Pharmacology, Pasteur Institute of Iran, Tehran, Iran; 2 Drug Design and Bioinformatics Unit, Department of Medical Biotechnology, Biotechnology Research Center, Pasteur Institute of Iran, Tehran, Iran; 3 Department of Pharmacology and Biochemistry, School of Medicine, Shahed University, Tehran, Iran

**Keywords:** Epilepsy, Drug screening, Pentylenetetrazole (PTZ), Maximal electroshock (MES)

## Abstract

**Background:**

Resistance to antiepileptic drugs and the intolerability in 20-30% of the patients raises demand for developing new drugs with improved efficacy and safety. Acceptable anticonvulsant activity, good tolerability, and inexpensiveness of docosahexaenoic acid (DHA) make it as a good candidate for designing and development of the new anticonvulsant medications.

**Methods:**

Ten DHA-based molecules were screened based on *in silico* screening of DHA-like molecules by root-mean-square deviation of atomic positions, the biological activity score of Professional Association for SQL Server, and structural requirements suggested by pharmacophore design. Anticonvulsant activity was tested against clonic seizures induced by pentylenetetrazole (PTZ, 60 mg/kg, i.p.) and tonic seizures induced by maximal electroshock (MES, 50 mA, 50 Hz, 1 ms duration) by intracerebroventricular (i.c.v.) injection of the screened compounds to mice.

**Results:**

Among screened compounds, 4-Phenylbutyric acid, 4-Biphenylacetic acid, phenylacetic acid, and 2-Phenylbutyric acid showed significant protective activity in pentylenetetrazole test with ED_50_ values of 4, 5, 78, and 70 mM, respectively. In MES test, shikimic acid and 4-tert-Butylcyclo-hexanecarboxylic acid showed significant activity with ED_50_ values 29 and 637 mM, respectively. Effective compounds had no mortality in mice up to the maximum i.c.v. injectable dose of 1 mM.

**Conclusion:**

Common electrochemical features and three-dimensional spatial structures of the effective compounds suggest the involvement of the anticonvulsant mechanisms similar to the parent compound DHA.

## INTRODUCTION

Epilepsy is the third most frequent neurologic disorder after cerebrovascular disease and dementia[1]. Nowadays, many old and new antiepileptic drugs (AEDs) are available. However, almost one-third of the epileptic patients remain unmanageable and refractory to AEDs[^2^]. Meanwhile, most of epileptic patients experience serious central and peripheral adverse effects of AEDs. Hence, there is a substantial need to find new AEDs with higher efficacy and less adverse effects. This has provided impetus to identify new AEDs by target-based and mechanism-oriented design.

Docosahexaenoic acid (DHA) exists in the evolution for more than 600 million years as component of cell membranes with high electrical activity [3]. DHA is a long chain polyunsaturated fatty acid (PUFA) with unique conformational characteristics and several functional roles in excitable membranes including neural cells. DHA and other PUFAs have beneficial effects on epileptic seizures [4-6]. Modulation of Na^+^, K^+^ and Ca^2+^ channels as well as gamma-amino butyric acid (GABA) neurotransmission are suggested to be involved in the anticonvulsant action of PUFAs including DHA[5]. Therefore, DHA is an exquisitely interesting target compound for development of new AEDs.

Root-mean-square deviation (RMSD) is a model, measuring the structural similarities of the chemicals in three-dimensional feature, by optimal rigid body superposition[7]. Computer Aided Drug Design is a very useful tool to maximize the efficacy and characterizations of new drug candidates[8,9]. The ligand-based method is an indirect approach for finding pharmacologically-active compounds when the experimental three-dimensional structure of the target molecule is not available[10,11]. Since no specific receptor has been recognized for antiepileptic effects so far, we used the pharmacophore method as a ligand-based drug design strategy to find DHA-like active molecules.

Maximal electroshock (MES) test is an animal model of primary generalized tonic seizures and provides identification of compounds which prevent seizure distribution. On the other hand, pentylenetetrazole (PTZ) test is an animal model of generalized myoclonic seizures and identifies compounds which primarily increase seizure threshold [12]. These methods are among the first screening tests used for decades in discovery of the new AEDs [12].

In the present study, 10 DHA-like compounds were screened by bioinformatics tools. Thereafter, anticonvulsant activity of the compounds was examined by MES and PTZ seizure tests in mice.

## MATERIALS AND METHODS

### Similarity search and *in silico* screening

Similarity search was performed to obtain collection of DHA-like molecules. The structure of DHA was uploaded into Prediction of Activity Spectra for Substances (PASS) and the threshold of ≥95% with no filter, used as options[13]. PASS is a software product, designed as a tool for evaluating the general biological potential of an organic drug-like molecule. PASS provides simultaneous predictions of many types of biological activity based on the structure of organic compounds. Thus, PASS can be used to estimate the biological activity profiles for virtual molecules, prior to their chemical synthesis and biological testing[14]. Activity tables provide Pa and Pi. Pa (probability “to be active”) estimates the chance that the studied compound is belonging to the sub-class of active compounds (resembles a sub-set of “actives” in PASS training set). Pi (probability “to be inactive”) estimates the chance that the studied compound is belonging to the sub-class of inactive compounds (resembles a sub-set of “inactives” in PASS training set). Robustness of PASS algorithm means that PASS provides reasonable estimates of structure-activity relationships despite of incompleteness (or some errors in data) of PASS training set[14].

Hyperchem (version 8.0.7) was used for adding hydrogen to each screening molecule and running their molecular dynamics computation with default options. The RMSD was calculated based on heavy atoms of the compounds compared to the DHA molecule. In order to obtain the biological activity spectrum (BAS) of the anticonvulsant activity, three-dimensional Mol files of DHA-like molecules were uploaded to PASS. The structures and characteristics of these molecules were used for selection of final compounds. In PASS, BAS of a chemical compound is the set of different types of biological activity that reflect the results of the compound’s interaction with various biological entities. BAS represents the “intrinsic” property of a substance depending only on its structure and physical-chemical characteristics.

### Pharmacophore design

As no specific receptor is identified for DHA, in the absence of the receptor structure, the ligand-based strategy produces pharmacophore models from a set of known ligands. This approach considers the conformational flexibility of the ligands. We choose DHA, eicosapentaenoic acid, and hexadecatrienoic acid as the training set for our pharmacophore. All compounds were generated by simplified molecular-input line-entry system code downloaded from PubChem and generated in the Chemsketech (Version 12.0). After adding molecules in LigandScout (Version 3.03b)[15, 16], conformations of each molecule were generated with 10.0 unit energy window and 30000 maximum number of generated conformations, and all other options, set as default.

The ligands were then aligned and superimposed by using an on-the-fly conformation generator of the LigandScout. After the alignment step, 10 pharmacophore models were generated by the pharmacophore elucidation algorithm. The models were ranked by scores based on the relative pharmacophore fit scores and the number of matching pharmacophore features. Pharmacophore models were validated by extent of fitness of the standard AEDs tiagabine and topiramate in the pharmacophores. The pharmacophore in which tiagabine and topiramate showed best fitting value was selected as final pharmacophore. Then The RMSD of the pharmacophore alignment was normalized.

### Selection of the lead compounds

DHA-like molecules are screened in the pharmacophore by importing them as a test set. The pharmacophore fit value 70 was considered as the cut off for screening. The higher pharmacophore fit scores (e.g. 80-100) results in a few compounds with no desired activity. In low value pharmacophore fit score of 50, many of compounds with capacity of anticonvulsant effect did not pass the pharmacophore criteria. Therefore, value 70 was selected as cut-off for the screening of compounds. Subsequently, some atoms and bonds in the selected molecules were manipulated by Chemsketech software by considering visual match of the molecules to pharmacophore, and different groups of the molecules were built. Thereafter, these were again screened on the pharmacophore. These processes repeated several times to find the most fitted molecules to the pharmacophore. Finally, compounds with the highest pharmacophore fit score were selected. In order to check potential side effects regarding to the off-targets, all possible targets for DHA and the selected compounds with the highest pharmacophore fit score were examined in Drug Bank database.

### Chemicals

Pentylenetetrazole, phenylacetic acid and 2-Phenylbutyric acid were purchased from Sigmaaldrich (Germany). 3,4-Dimethoxyphenylacetic acid, 4-Phenylbutyric acid, 2-Thiophene ethanol, Fluorene-9-carboxylic acid, 1-Adamantanecarboxylic acid, 4-Biphenylacetic acid, 4-tert-Butylcyclohexane-carboxylic acid, and shikimic acid were obtained from Merck (Germany).

### Animals

Male NMRI mice 5-6 week old, weighing 20-28 g, bred in the animal facility of Pasteur Institute of Iran, were used in this study. They were housed in groups of 12 mice in polypropylene cages with automatically controlled 12 h light/dark cycle, with lights turned on at 7:00 am, at a constant temperature of 22±2°C and a relative humidity of 60-70%. All animals were freely accessed to standard diet and drinking water. All experiments were conducted during light phase and performed in accordance with the guidelines of the Institutional Animals Ethics Committee of Pasteur Institute and conformed the European Communities Council Directive of 24 November 1986 (86/609/EEC).

### Drugs administration

All compounds were prepared freshly before administration. The compounds were dissolved in DMSO and then were diluted in artificial cerebrospinal fluid consisting (in mM) 124.0 NaCl, 25 NaHCO_3_, 10 D-glucose,4.4 KCl, 2 MgSO_4_, 1.25 KH_2_PO_4_, and 2 CaCl_2_. The final concentration of DMSO was maintained at 10%. The pH of the solutions was adjusted at the range of 7.2-7.4. Doses of the compounds were selected based on the preliminary experiments conducted in our laboratory.

DHA-like molecules were administered by intracerebroventricular (i.c.v.) route according to the method described earlier[6]. Briefly, a 3.5-mm-long needle (27 Gauge) was connected to a 10-μL Hamilton microsyringe. The site of injection was 2 mm lateral to the midline on a line drawn through the anterior base of the ears and 3.5 mm in depth from the surface of skull skin. The solution was injected at a rate of 10 µL/10 s and the needle left in place for 60 s after injection.

### Lethality test

Compounds were injected i.c.v. at the dose of 1 M. Then the mice were observed for 72 h, and the rate of mortality was recorded.

### Pentylenetetrazole seizure test

Fifteen minutes after i.c.v. injection of the compounds, PTZ (60 mg/kg) was injected to the mice intraperitoneally (i.p.). This time period was selected based on the time course of DHA anticonvulsant activity in mice[6]. The occurrence of colonic seizures was monitored in animals for 30 min.

### Maximal electroshock seizure test

Fifteen minutes after i.c.v. injection of the compounds, an electrical stimulus (50 Hz, 50 mA, 1 s duration) was delivered to the mice through ear-clip electrodes[6] using a stimulator apparatus (MGH-777, Development of Electronic Industry, Iran). The number of animals that demonstrated hind limb tonic extension (HLTE) was recorded.

### Data analysis

Statistical analysis was performed by SPSS for Windows software version 16.0. Data obtained from the seizure tests were expressed as percentage of the protected mice. Fisher’s exact test was used to analyze the data. The dose of the drugs that produced anticonvulsant effect in 50% of the animals (ED_50_), and its associated 95% confidence interval, was calculated by log-Probit analysis.

## RESULTS

### Lead compounds

**Fig. 1:**
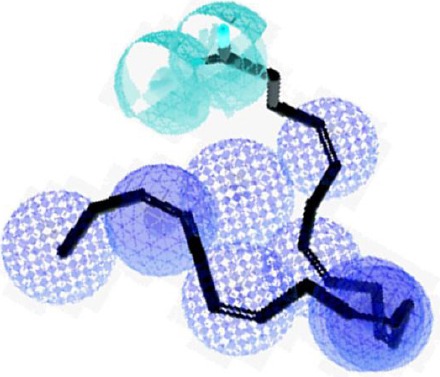
Docosahexaenoic acid (DHA) molecule (solid lines) inscribed with DHA-like molecule pharmacophore (spheres). Pharmacophore features are color-coded as hydrophobic feature (blue sphere), and electron donor group features are green sphere.

Sixty-Nine compounds were found in PubChem with the same core structure of DHA. All of the compounds had common carboxyl functional groups with a fatty acid tail, with different carbon numbers and various *cis* or *Trans* bond positioning. The three-dimensional structure of these compounds was optimized using the molecular dynamic simulation process, and normalized RMSD value measured. Among 69 compounds, 57 molecules had normalized RMSD value below 0.4 compared to DHA. PASS server prediction showed that 29 compounds have biological (anticonvulsant) activity with Pa value in the range of 0.5-0.7. Figure 1 shows the DHA molecule inscribed with the designed pharmacophore model. The pharmacophore model consists of hydrophobic features and electron donor groups in specific spatial arrangement. Ten best fitted molecules with the highest pharmacophore fit score were selected as lead compounds. The two-dimensional structure of the selected compounds is shown in Table 1. By searching in Drug Bank data base, no relevant off-target for DHA, and the lead compounds was found.

**Table 1:** Chemical structure of the lead compounds with the highest pharmacophore fit score

No.	Chemical name	Two-dimentional structure	Pharmacophore fit value
01	3, 4-Dimethoxyphenylacetic acid	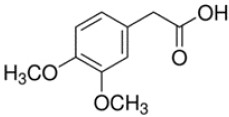	47.90
02	4-Phenylbutyric acid	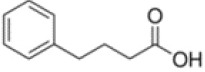	48.32
03	Fluorene-9-carboxylic acid	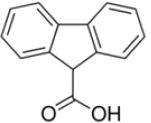	53.61
04	2-Thiophene ethanol	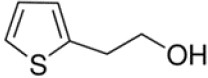	0.00
05	1-Adamantanecarboxylic acid	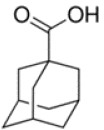	38.64
06	4-Biphenylacetic acid	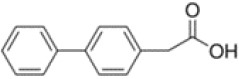	53.92
07	4-tert-Butylcyclohexanecarboxylic acid	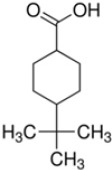	45.29
08	Shikimic acid	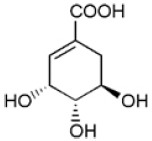	38.78
09	Phenylacetic acid	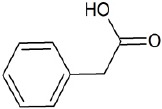	48.16
10	2-Phenylbutyric acid	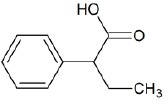	53.14

### Anticonvulsant activity

At the doses were used, the selected compounds, did not induce any abnormal behavior or mortality in animals. Among 10 compounds, 4-Phenylbutyric acid, 4-Biphenylacetic acid, phenylacetic acid, and 2-Phenylbutyric acid showed significant anticonvulsant activity against seizures induced by PTZ and ED_50_ values of 4 mM (CI=0.0-7.0), 5 mM (CI=3.0-5.0), 78 mM (CI=46.0-156.0) and 70 mM (CI=25.0-144.2) were obtained, respectively (Table 2). In MES test, shikimic acid and 4-tert-Butylcyclohexanecarboxylic acid showed significant activity with ED_50_ values of 29 mM (CI=0.0-66.0) and 637 mM (CI=418.0-890.0), respectively (Table 3).

**Table 2:** Effective compounds against clonic seizures induced by pentylenetetrazole in mice

Compound	Dose (M)	Protection (%)	N	ED_50_ (mM) and 95% CI
Control	0	0	10	-
4-Phenylbutyric acid	0.01	73.3^a^	30	4.0 (0.0-7.0)
	0.001	50^b^	20	
	0.0001	25	20	
4-Biphenylacetic acid	0.01	85^a^	20	5.0 (3.0-5.0)
	0.0075	50	20	
	0.001	35	20	
	0.0001	15	20	
Phenylbutyric acid	0.1	57.5^a^	40	78.0 (46.0-156.0)
	0.01	30	30	
	0.001	20	10	
	0.0001	0	10	
2-Phenylbutyric acid	0.1	56.6^a^	30	70.0 (25.0-144.2)
	0.01	40^b^	30	
	0.001	30	30	
	0.0001	0	10	

a *P*<0.01 and

b *P*<0.05 vs. control group by Fisher’s exact probability test. N, number of mice used for each experiment.

**Table 3:** Effective compounds against tonic seizures induced by MES in mice

Compound	Dose (M)	Protection (%)	N	ED_50_ (mM) and 95% CI
Control	0	0	10	-
4-tert-Butylcyclohexane carboxylic acid	0.1	73.3^a^	30	29.0 (0.0-66.0)
	0.01	46.6^b^	30	
	0.001	37.5	40	
Shikimic acid	1	70.0^a^	30	637.0 (418.0-890.0)
	0.5	43.3^b^	30	
	0.1	20.0	20	
	0.01	0	10

a *P*<0.01 and

b *P*<0.05 vs. control group by Fisher’s exact probability test. N, number of mice used for each experiment.

## DISCUSSION

In our study, 4 compounds, 4-Phenyl butyric acid, 4-Biphenyl acetic acid, phenyl acetic acid, and 2-Phenyl butyric acid showed significant anticonvulsant activity in PTZ seizure test. In addition, 2 compounds, 4-tert-Butylcylclohexane carboxylic, and shikimic acid had significant anticonvulsant effect in MES model.

DHA and other PUFA have beneficial effect on epileptic seizures[5,6]. DHA molecule has a carboxylic functional group with a fatty acid chain. This carboxylic functional group can fit in a specific pharmacophore site in the receptor. Moreover, the carbon chain, which has several unsaturated bonds can bend and rotate to form a special three dimensional structure to fit the receptor. The pharmacophore site and the unique three dimensional structures give a distinctive feature to DHA as a remarkable model in screening effective drugs.

The proper pharmacophore modeling is the first and critical step in bioinformatics approach to design new effective medications. In this phase, several pharmacophore models are designed. Afterward, among all the pharmacophores, the best one is validated by standard drugs[17-19]. There are evidences that unsaturated fatty acids such as DHA are able to modulate voltage dependent K^+^ channels[4,5]. Meanwhile, the standard AEDs tiagabine and topiramate inhibit epileptiform bursting by modulation of potassium channels[20,21]. Therefore, tiagabine and topiramate were selected as the reference molecules to validate the designed pharmacophore. Both drugs fitted well in the pharmacophore, and the fitting value greater than 70% was obtained for both.

The anticonvulsant compounds, 4-Phenylbutyric acid, 4-Biphenylacetic acid, phenylacetic acid, and 2-Phenylbutyric acid, share a phenyl group in their structure. This functional group is present in some of the previous well-known AEDs, such as phenytoin and phenobarbital. However, it seems this construction is functional when it is not enclosed in the center of the molecule. For instance, although phenyl group exists in 3,4-Dimethoxyphenylacetic acid, it is hidden in the center of the molecule, which causes the molecule to be inactive as an anticonvulsant. On the other side, shikimic acid and 4-tert-Butylcyclohexancarboxylic acid have pyramid-like structures with carboxylic acid located at the top of the molecule. This structure which is able to inhibit MES-induced tonic seizures is unique and not seen in current AEDs.

In PTZ test, DHA possesses dose-dependent protective effect in mice with ED_50_ of 0.13 µM in i.c.v administration[6]. In the present study, ED_50_ value of the effective compounds in PTZ test was much higher than the parent compound DHA. This means that the screened compounds in our study are far less potent compared to DHA. However, DHA is very sensitive to oxidation and is unstable. In addition, DHA has a very flexible and multi-conformational structure while the screened effective compounds are more structurally-stable and therefore possess more drug-like properties for further studies. Furthermore, DHA is not able to inhibit tonic seizures in the MES model[6]; whereas the screened molecules in our study, shikimic acid and 4-tert-Butylcyclohexancarboxylic acid, have acceptable anticonvulsant activity in this seizure model.

It is well known that drugs effective in MES test act by blocking voltage-dependent sodium channels, or by blocking glutamatergic excitation[22]. On the other hand, seizures induced by PTZ, can be inhibited by reduction of T-type Ca^2+^ channels, or enhancement of GABAergic transmission[22]. Therefore, in addition to modulation of K^+^ channels, these mechanisms might also be involved in the observed protective effect of the screened molecules.

The present study is the first report on anticonvulsant activity of 4-Phenylbutyric acid, 4-Biphenylacetic acid, phenyl acetic acid, 2-Phenylbutyric acid, shikimic acid and, 4-tert-Butylcyclohexane carboxylic acid. These compounds are not unknown molecules in medical literature. 4-Phenylbutyric acid was identified more than 30 years ago as an ammonia scavenger, and FDA approved its application for treatment of the urea cycle disorder. This small chemical chaperone inhibits endoplasmic reticulum stress and is effective against many of endoplasmic reticulum stress-mediated diseases, such as various types of cancers, as well as neurodegenerative disorders such as Parkinson’s disease. Furthermore, it imparts its anti-inflammatory activity by reducing inflammatory cytokines[23]. Meanwhile, phenylacetic acid is the metabolite of 4-Phenylbutyric acid and is believed to partially exert the pharmacological actions of the parent drug. The 4-Biphenyl acetic acid and phenylacetic acid belong to the category of non steroidal anti-inflammatory drugs that inhibit platelet aggregation and have radical scavenging and anti-thrombotic activity[24]. The other interesting anticonvulsant compound, shikimic acid, is a natural organic compound with antioxidant, anticoagulant, antithrombotic, antibacterial, anti-inflammatory, antipyretic, and analgesic activities[25]. It is generally utilized as a starting material for industrial synthesis of the antiviral oseltamivir, a drug against influenza virus[25]. Thus, it seems that these compounds are promising for further studies on their possible indication as therapeutic molecules.

In conclusion, we found 10 anticonvulsant molecules by computer-aided drug design. Six of them showed acceptable anticonvulsant activity in the main preliminary screening tests, PTZ and MES. Efficacy of the compounds in other models of seizures and epilepsy such as kindling and 6Hz model of psychomotor seizures are needed to reveal their possible clinical merit.
